# IL-1β and IL-6 modulate apolipoprotein E gene expression in rat hepatocyte primary culture

**DOI:** 10.1155/S0962935192000498

**Published:** 1992

**Authors:** Agnes Ribeiro, Marise Mangeney, Isabelle Bernard, Jean Chambaz, Francisco Delers

**Affiliations:** 1 CNRS URA 1283 CHU Saint Antoine 27 rue de Chaligny Paris 75012 France; 2 Laboratoire des Protéines de la Réaction Inflammatoire Faculté de Médecine 45 rue des Saints Péres Paris Cedex 06 Paris 75270 France

## Abstract

Incubation of rat hepatocytes in primary culture with IL-1β at a concentration of 2.5 units/ml resulted in an increase (+80%) in the amount of apoE mRNA without any effect upon apoE synthesis. IL-6 at a low concentration (10 units/ml) induced a decrease (−35%) in the amount of apoE mRNA, but increased apoE synthesis (+28%). No effect was observed with higher concentrations of IL-1β (10 units/ml) or IL-6 (100 units/ml). These results suggest that inflammatory cytokines IL-1β and IL-6 modulate the expression of apoE gene in cultured rat hepatocytes, at a concentration that does not induce the acute phase response.


Mediators of Inflammation, 1, 329–333 (1992)

### IL-1$\beta$ and IL-6 modulate apolipoprotein E gene expression in rat hepatocyte primary culture

Agnes Ribeiro,[1] Marise Mangeney,[1] Isabelle Bernard,[2] Jean Chambaz[1] and Francisco Delers[1,2,CA]

[1]CNRS URA 1283, CHU Saint Antoine, 27 rue de Chaligny, Paris 75012, France;

[2]Laboratoire des Protéines de la Réaction Inflammatoire, Faculté de Médecine, 45 rue des Saints Pères, 75270 Paris Cedex 06, France

[CA] Corresponding Author

**Incubation of rat hepatocytes in primary culture with IL-1$\beta$ at a concentration of 2.5 units/ml resulted in an increase (+80%) in the amount of apoE mRNA without any effect upon apoE synthesis. IL-6 at a low concentration (10 units/ml) induced a decrease (−35%) in the amount of apoE mRNA, but increased apoE synthesis (+28%). No effect was observed with higher concentrations of IL-1$\beta$ (10 units/ml) or IL-6 (100 units/ml). These results suggest that inflammatory cytokines IL-1$\beta$ and IL-6 modulate the expression of apoE gene in cultured rat hepatocytes, at a concentration that does not induce the acute phase response.**

**Key words:** Acute phase response, Apolipoprotein-E, Interleukin-1$\beta$, Interleukin-6, Rat hepatocytes

### Introduction

Apolipoprotein-E (apoE) exerts an important biological role in lipid redistribution in many different organs due to its ability to bind to specific receptors. Although several cell types participate in the production of apoE,[1] plasma apoE is mainly synthesized by the liver in a highly regulated fashion. Both fasting[2] and an increased glucagon/insulin ratio[3] induce a specific increase in liver apoE mRNA and enhance apoE secretion by rat liver. On the other hand, we have observed a decrease of both apoE mRNA and apoE secretion by hepatocytes isolated from rats fed for 3 weeks on a fish oil diet. Such a decrease could be partly correlated to the insulin/glucagon ratio.[4]

Dramatic changes in the synthesis of a large series of plasma proteins are observed during a liver acute phase response[5] which is induced and modulated by a large set of cytokines, often in an antagonistic way. The availability of recombinant cytokines has allowed progress in the understanding of the cellular and molecular mechanisms involved in liver acute phase response (APR). In fact interleukin-6 (IL-6)[6] and interleukin-1$\beta$ (IL-1$\beta$),[7] which are mainly secreted by activated macrophages, have been shown to induce partial acute phase response in rat hepatocyte primary culture.[8] IL-6 binds to specific receptors and activates, via a transduction pathway which has not yet been elucidated, at least two nuclear factors (NF-IL-6), which bind specific DNA sequences (IL-6 RE), located in the 5′ flanking region of almost all the acute phase protein genes.[9,10] Similarly, IL-1$\beta$ exerts its effects by binding to a specific receptor, but its transduction pathway has not been clearly defined. IL-1$\beta$ exerts transcriptional effects which are mediated at the DNA level by an NF$\kappa$B transactivating factor.[11] The effects of IL-1$\beta$ are not restricted to the transcriptional level, since it has been shown that IL-1$\beta$ also modulates translation in liver cells.[12,13]

It has been reported that the apoE mRNA level is not modified during an acute phase response *in vivo*[14] or after induction with tumour necrosis factor alpha (TNF$\alpha$).[15] Recently IL-6, as well as TNF, has been shown to induce hepatic lipogenesis.[16,17] Individual cytokines induce a partial acute phase response (APR) whereas, *in vivo*, APR results from the combined effects of cytokines upon liver parenchymal cells. The aim of the present study was therefore to determine the effect of individual cytokines IL-1$\beta$ and IL-6 on apoE biosynthesis by rat hepatocytes in primary culture.

### Materials and Methods

*Materials:* Recombinant human (rh) IL-6 and IL-1$\beta$ were provided by Genzyme. Rh IL-6 had a specific activity of $4 \times 10^6$ units per mg and rh IL-1$\beta$ $5 \times 10^8$ units per mg. Minimal essential medium (MEM), antibiotics and foetal calf serum were obtained from Gibco. Protein A–Sepharose was obtained from Pharmacia. Insulin and dexamethasone were obtained from Sigma. Collagenase was obtained from Boehringer. [$^{35}$S]-methionine, [$\alpha^{32}$P]-dCTP, Nylon hybond $N^+$ filters and X-ray films were from Amersham International. Acrylamide, *N*,*N*′-methylenebisacrylamide (electrophor-

© 1992 Rapid Communications of Oxford Ltd esis grade) and molecular weight markers were obtained from Biorad. The nick-translation kit was obtained from BRL.

*Primary culture of hepatocytes:* Male Wistar rats (IFFA-CREDO, France) of weight 200 $\pm$ 20 g at 6 weeks were housed in individual cages and maintained on a 12 h light/dark cycle at controlled room temperature (20°C) and fed *ad libitum* with a standard diet. Sacrifice was performed at 09.00 h in the post-prandial period. Primary cultures of hepatocytes isolated by collagenase perfusion, were prepared as described previously.[4] Hepatocytes ($5 \times 10^6$ cells/dish) were plated in MEM containing antibiotics (kanamycin, penicillin, streptomycin and gentamicin) and supplemented with foetal calf serum (10% v/v) and 1 $\mu$M insulin. After 4 h, the medium was replaced for 18 h with MEM containing antibiotics and supplemented with 1 $\mu$M dexamethasone.

Albumin and alpha-2 macroglobulin (A2M) were determined in non-labelled culture media by electroimmunoassay as described.[18] All the results were expressed using arbitrary units relative to serial dilutions of inflammatory rat serum.

*Protein labelling and immunoprecipitation:* Hepatocytes were incubated for a further 24 h in the presence or in the absence of cytokines. Cells were washed three times with 2 ml methionine-free MEM and labelled with [$^{35}$S]-methionine (60 $\mu$Ci/dish; specific activity 1000 Ci/mol) in the same medium. After 3 h, the medium was collected and centrifuged at 4000 rpm for 10 min to remove cell debris. Cells were washed twice with cold 20 mM phosphate buffer, pH 7.5, containing 0.15 M NaCl, scraped from the dish into homogenization buffer: 20 mM Tris/HCl (pH 7.4), containing 5 mM EDTA, 1% (v/v) Triton X-100, 0.1% SDS and 1 mM phenylmethylsulphonyl fluoride, and then disrupted by rapid up and down pipetting. Lysates and medium samples were mixed with 0.2 volume of bovine-serum-albumin–Sepharose CL4B (5 mg of BSA per ml of gel) equilibrated in 25 mM Tris/HCl buffer (pH 7.4), 0.15 M NaCl, 5 mM EDTA, 1% (w/v) sodium deoxycholate and 1% (v/v) Triton X-100 (buffer A). The suspension was rotated for 2 h at 4°C, the gel was rapidly sedimented ($13\,000 \times g$, 1 min) and the supernatant was used for immunoprecipitation.[4]

*RNA analysis:* Total RNA was prepared from hepatocytes using the guanidium/phenol/chloroform method.[19] The total RNA content of cultured cells was measured by spectrophotometry and its integrity was assayed by agarose gel electrophoresis. Serial dilutions of total RNA were applied to nylon membranes and hybridization was carried out with cDNA probes labelled with [$\alpha^{32}$P]-dCTP (resulting specific radioactivity around $10^8$–$10^9$ cpm/$\mu$g cDNA) by the nick-translation technique. ApoE cDNA probe was obtained as described previously.[3] Alpha-2-macroglobulin, alpha-1 acid glycoprotein (AGP) and $\beta$-actin cDNA probes were kindly given by G. H. Fey, J. M. Taylor and M. Buckingham respectively. The hybridization solution contained 0.9 M NaCl, 0.09 M sodium citrate, 5 $\times$ Denhardt's solution, 0.1% w/v SDS, 50% v/v formamide and 100 $\mu$g/ml denatured herring sperm DNA. Following an 18 h incubation at 42°C, membranes were washed twice for 15 min each in 0.3 M NaCl, 0.03 M sodium citrate, 0.1% SDS at room temperature, and for 60 min at 42°C in the same solution without SDS. Following autoradiography, mRNA abundance was estimated by quantitative scanning densitometry using an LKB laser densitometer. The relative abundance of specific mRNA was calculated by reference to the content of $\beta$-actin mRNA.

*Statistical determinations:* Statistical significance of results was assessed using the Student's *t*-test.

### Results and Discussion

Specific parameters of the acute phase response in rat hepatocytes in primary culture were checked in order to: (1) avoid a previous inflammatory reaction in rats; (2) avoid stimulation by cytokines resulting from contamination of the hepatocyte preparation by endothelial or Kupffer cells. Indeed, activated Kupffer and endothelial cells can produce cytokines and IL-1$\beta$, and therefore can induce IL-6 synthesis by these cell types; and (3) assess the responsiveness of the hepatocytes in primary culture to cytokines. Control and IL-1$\beta$ stimulated cultures secreted no detectable alpha-2 macroglobulin (A2M), which eliminated contamination of cultured hepatocytes by Kupffer and endothelial cells (Figure 1). The responsiveness of hepatocytes to IL-6 was demonstrated by the increase in A2M mRNA level and secretion, and by the decrease in those of albumin. Furthermore, the increase in alpha-1-acid glycoprotein (AGP) mRNA in IL-1$\beta$ treated cultures established the responsiveness of hepatocytes to IL-1$\beta$ (Figure 1).

As shown in Figure 2A, incubation of hepatocytes with a low concentration of IL-6 (10 units/ml) for 24 h resulted in a 30% decrease in the apoE mRNA level, which was not paralleled by a similar modification in apoE synthesis. On the contrary, we observed a 28% increase in the incorporation of methionine into newly synthesized apoE (Figure 3). Furthermore, a 10-fold higher concentration of IL-6 did not change the apoE mRNA level, but induced a 23% decrease in apoE synthesis by hepatocytes. These discrepancies

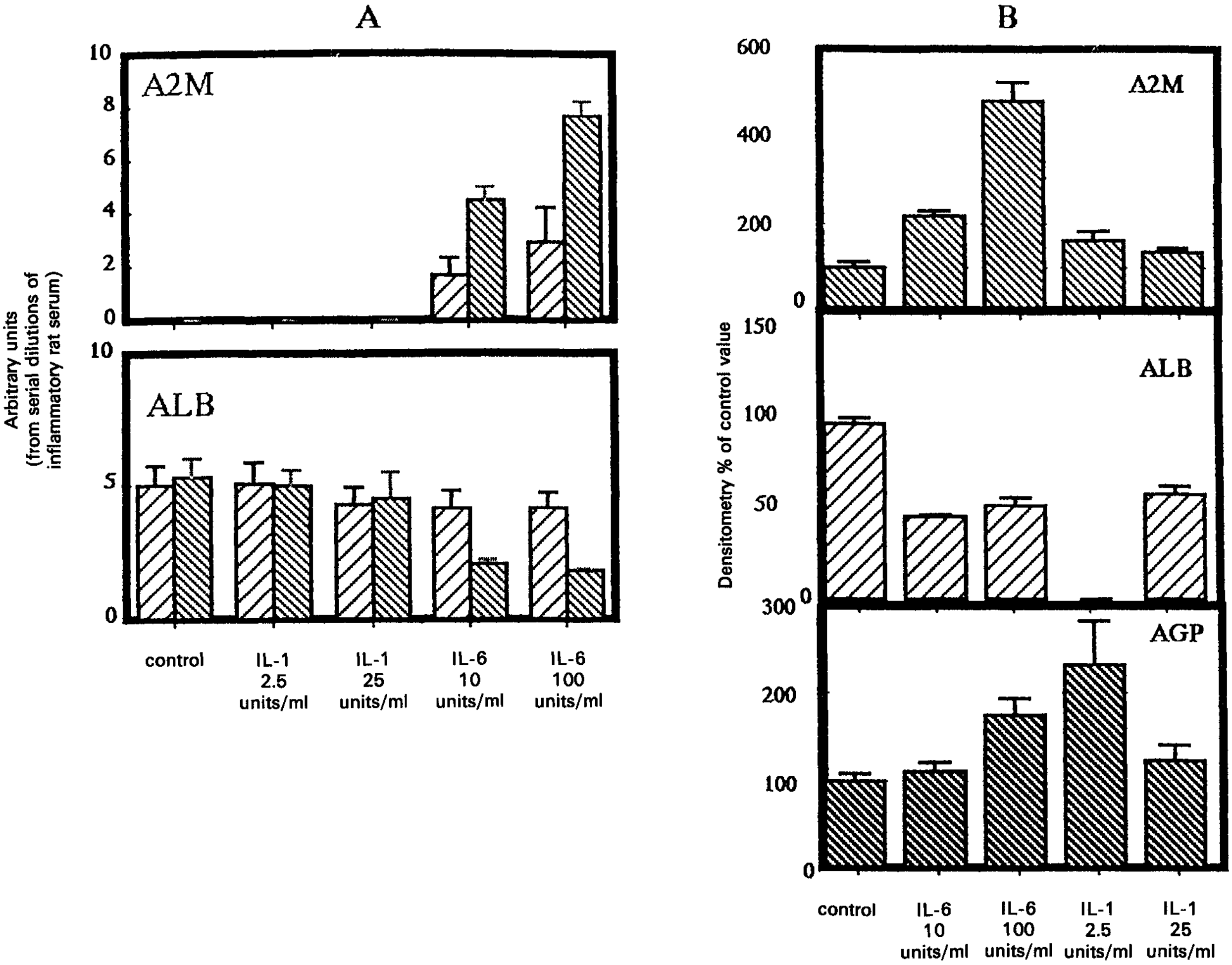

FIG. 1. Effects of IL-6 and IL-1$\beta$ on alpha-2-macroglobulin (A2M), alpha-1 acid glycoprotein (AGP) and albumin (ALB) synthesis (panel A) and mRNA levels (panel B) in rat hepatocyte primary culture. Panel A: Rat hepatocytes in primary culture, kept in culture for 24 h, were incubated at 37°C with the indicated doses of IL-1$\beta$ and IL-6 for 24 h (widely hatched bars) or 48 h (closely hatched bars). Each result is the mean ($\pm$SD) of triplicate determinations in three different experiments. Panel B: Serially diluted (from 4 $\mu$g to 0.5 $\mu$g) cytoplasmic RNAs from hepatocytes stimulated for 24 h with cytokines, as indicated, were probed with [$^{32}$P]cDNA probes encoding for A2M, AGP and albumin. Specific mRNA levels relative to those of $\beta$-actin mRNA are expressed as arbitrary units determined by densitometry analysis of the autoradiogram. $\beta$-actin mRNA remained stable during IL-1$\beta$ or IL-6 treatment. Each result is the mean ($\pm$SD) of triplicate determinations in three different experiments.

between mRNA level and protein synthesis rate could be explained by an effect of IL-6 on the translation machinery. Such an effect has already been observed in the liver during the acute phase response.[20]

In addition, it can be seen from Figure 3 that IL-6 treatment of hepatocytes modified the ratio between apoE isoforms in favour of the lower form. The low Mr/high Mr ratio of secreted apoE was modified from 1.3 in controls to 1.9 and 2.5 in cultures stimulated with 10 and 100 units/ml of IL-6 respectively, while it remained unchanged (3.2) for intracellular apoE. This relative increase in the low Mr apoE isoform could be related to modifications in oligosaccharide processing, as already observed for alpha-1 protease inhibitor during IL-6 induced acute phase response.[21] However, carbohydrate units are not processed in the same manner for both proteins since apoE is an O-glycosylated protein,[22] whereas alpha-1 protease inhibitor is an *N*-glycosylated protein. Alternatively, these variations in apoE-Mr may result from a modification of the kinetics of apoE intracellular transport and/or of its routing along the secretory pathway, as it has been previously observed in rabbit liver for C reactive protein (CRP).[23]

A low concentration of IL-1$\beta$ (2.5 units/ml) induced a 80% increase in apoE mRNA content in hepatocytes, whereas no change was observed with a higher concentration of IL-1$\beta$ (Figure 2B). ApoE synthesis was neither quantitatively nor qualitatively modified whatever the concentration of IL-1$\beta$. Similarly, the ratio between low and high Mr isoforms of secreted and intracellular apoE remained stable at 1.32 and 3.2 respectively, whatever the IL-1$\beta$ concentration (Figure 4). The discrepancy between apoE mRNA level and the rate of protein synthesis could result from a translational

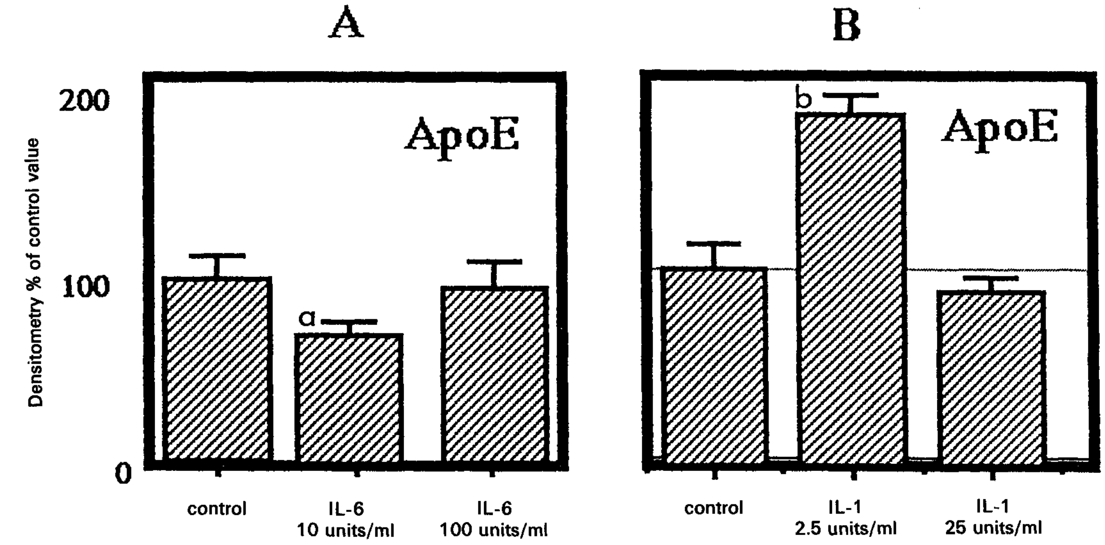

FIG. 2. Effect of IL-6 (panel A) and IL-1$\beta$ (panel B) on apoE mRNA in rat hepatocytes in primary culture. Serially diluted (from 4 $\mu$g to 0.5 $\mu$g) cytoplasmic RNAs from hepatocytes stimulated for 24 h with cytokines, as indicated, were probed with [$^{32}$P]cDNA encoding for apolipoprotein E (ApoE). ApoE mRNA levels are expressed, in relation to those of $\beta$-actin mRNA, as arbitrary units determined by densitometry analysis of the autoradiogram. Results are means ($\pm$SD) of three experiments performed in triplicate. (a) $p < 0.05$; (b) $p < 0.01$.

inhibition of apoE expression by IL-1$\beta$ at a low concentration. In fact, although IL-1$\beta$ has been shown to increase the translation rates of CRP[12] and ferritin[13] mRNA in human hepatoma cells, an inhibitory effect of IL-1$\beta$ on mRNA translation has not yet been described.

The different effects of low and high concentrations of IL-1$\beta$ and IL-6 on hepatocyte apoE mRNA could result from the interaction of both IL-1$\beta$ and IL-6 with high and low affinity receptors involving two different signal transduction pathways. Such types of cytokine receptors have been described for IL-6 in HepG2[24] and for IL-1$\beta$ in Th2 cells.[25]

The treatment of rat hepatocytes with 100 units/ml of IL-6 or with 25 units/ml of IL-1$\beta$, alone, has been shown to induce maximal effects in terms of acute phase response.[8] At such concentrations of IL-1$\beta$ and IL-6, we did not observe any change in either the apoE mRNA level or the protein synthesis rate by rat hepatocytes. These results are in agreement with previous observations during the late acute phase response *in vivo*.[14,15] In contrast, our

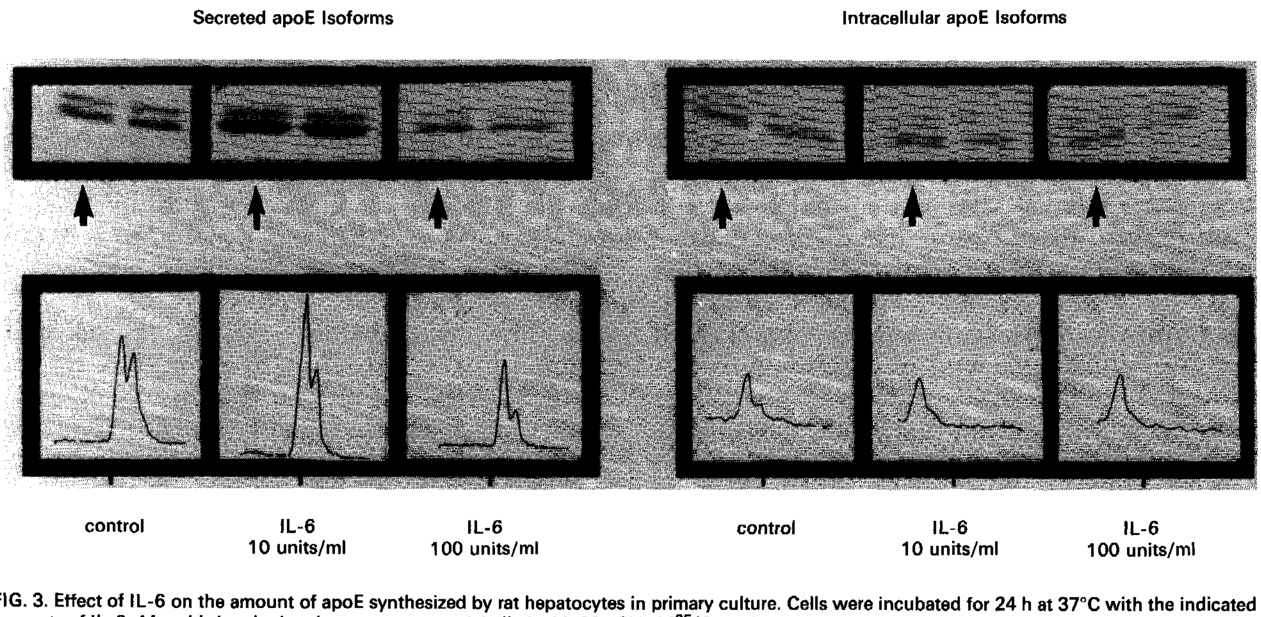

FIG. 3. Effect of IL-6 on the amount of apoE synthesized by rat hepatocytes in primary culture. Cells were incubated for 24 h at 37°C with the indicated amounts of IL-6. After this incubation, hepatocytes were labelled with 60 $\mu$Ci/ml [$^{35}$S]-methionine for 3 h. ApoE was recovered by immunoprecipitation from media and cell lysates and submitted to sodium dodecyl sulphate/polyacrylamide gel electrophoresis and fluorography. The relative amounts of apoE isoforms were then quantified by densitometry. Three experiments were performed in triplicate. Autoradiogram of a representative experiment was shown. The quantification by densitometry scan concerned the lane indicated by an arrow.

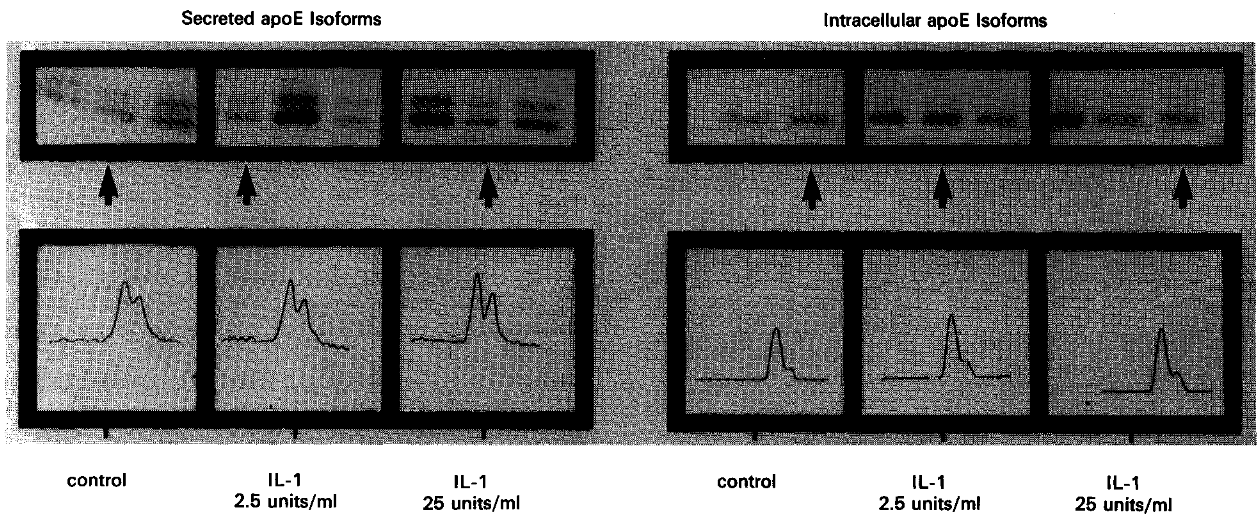

FIG. 4. Effect of IL-1$\beta$ on the amount of apoE synthesized by rat hepatocytes in primary culture. Cells were incubated for 24 h at 37°C with the indicated amounts of IL-1. After this incubation, hepatocytes were labelled with 60 $\mu$Ci/ml [$^{35}$S]-methionine for 3 h. ApoE was recovered by immunoprecipitation from media and cell lysates and submitted to sodium dodecyl sulphate/polyacrylamide gel electrophoresis and fluorography. The relative amounts of apoE isoforms were quantified by densitometry. Three experiments were performed in triplicate. Autoradiogram of a representative experiment was shown. The quantification by densitometry scan concerned the lane indicated by an arrow.

present results appear to indicate that lower levels of IL-1$\beta$ and IL-6 control apoE biosynthesis by rat hepatocytes in primary culture, at the level of apoE mRNA for both cytokines and at the post-transcriptional level for IL-6.

### References

1. Mahley, RW. Apolipoprotein E: Cholesterol transport protein with expanding role in cell biology. *Science* 1988; **241**: 622–630.
2. Davis RA, Dluz SM, Leighton JK, Brengaze VA. Increased translatable mRNA and decreased lipogenesis are responsible for the augmented secretion of lipid deficient apolipoprotein E by hepatocytes from fasted rats. *J Biol Chem* 1989; **264**: 8970–8977.
3. Mangeney M, Cardot P, Lyonnet S, *et al.* Apolipoprotein gene expression in rat liver during development in relation to insulin and glucagon. *Eur J Biochem* 1989; **181**: 225–230.
4. Ribeiro A, Mangeney M, Cardot P, *et al.* Effect of dietary fish oil and corn oil on lipid metabolism and apolipoprotein gene expression by rat liver. *Eur J Biochem* 1991; **196**: 499–507.
5. Birch H, Schreiber G. Transcriptional regulation of plasma protein synthesis during inflammation. *J Biol Chem* 1986; **261**: 8077–8080.
6. Gauldie J, Richards C, Harnish D, Lansdorp P, Baumann H. Interferon$\beta$2/B-cell stimulatory factor type2 shares identity with monocyte-derived hepatocyte-stimulating factor and regulate the major acute phase protein response in liver cells. *Proc Natl Acad Sci USA* 1987; **84**: 7251–7255.
7. Dinarello CA. Interleukin-1. *Rev Infect Dis* 1984; **6**: 51–95.
8. Andus T, Geiger T, Hirano T, *et al.* Regulation of synthesis and secretion of major rat acute phase proteins by recombinant human interleukin-6 (BSF-2/IL6). *Eur J Biochem* 1988; **173**: 287–293.
9. Oliviero S, Cortese R. The human haptoglobin gene promoter: interleukin-6 responsive element interact with a DNA-binding protein induced by interleukin-6. *EMBO* 1989; **J.8**: 1145–1151.
10. Poli V, Cortese R. Interleukin-6 induces a liver specific nuclear protein that binds to the promoter of acute-phase genes. *Proc Natl Acad Sci USA* 1989; **86**: 8202–8206.
11. Edbrooke MR, Burt DW, Cheshire JK, Woo P. Identification of cis-acting sequences responsible for phorbol ester induction of human amyloid A gene expression via a nuclear factor kB-like transcriptional factor. *Mol Cell Biol* 1989; **9**: 1908–1916.
12. Ganter U, Arcone R, Toniatti C, Morrone G, Ciliberto G. Dual control of C-reactive protein gene expression by interleukin-1 and interleukin-6. *EMBO* 1989; **J.8**: 3773–3779.
13. Rogers JT, Bridges K, Durmowicz GP, Glass J, Auron PE, Munro HM. Translational control during the acute phase response. Ferritin synthesis in response to interleukin-1. *J Biol Chem* 1990; **265**: 14572–14578.
14. Guo-Fen T, De Jong F, Apostopoulos J, *et al.* Effect of acute inflammation on rat apolipoprotein mRNA levels. *Inflammation* 1987; **11**: 241–251.
15. Delers F, Mangeney M, Raffa D, *et al.* Changes in rat liver mRNA for alpha1-acid-glycoprotein, apolipoprotein E, apolipoprotein B and beta-actin after mouse recombinant tumor necrosis factor injection. *Biochem Biophys Res Comm* 1989: **161**: 81–88.
16. Feingold KR, Serio MK, Adi S, Moser AH, Grunfeld C. Tumor necrosis factor stimulates hepatic lipid synthesis and secretion. *Endocrinology* 1989; **124**: 2336–2342.
17. Grunfeld C, Adi S, Soued M, Moser AH, Fiers W, Feingold KR. Search for mediators of the lipogenic effects of tumor necrosis factor: potential role for interleukin-1. *Cancer Res* 1990; **50**: 4233–4238.
18. Guillouzo A, Delers F, Clement B, Bernard N, Engler R. Long term production of acute phase proteins by hepatocytes cocultured with another liver cell type in serum free medium. *Biochem Biophys Res Comm* 1984; **120**: 311–317.
19. Chomczynski P, Sacchi N. Single step method of RNA isolation by acid guanidium thiocyanate-phenol-chloroform extraction. *Anal Biochem* 1987; **162**: 156–159.
20. Piccoletti R, Aletti MG, Bernelli-Zazzera A. Inflammation associated events in liver nuclei during acute phase reaction. *Inflammation* 1986; **10**: 109–117.
21. Mackiewicvz A, Kushner I. Interferon$\beta$2/B-cell stimulating factor2/interleukin-6 affects glycosylation of acute phase proteins in human hepatoma cells. *Scand J Immunol* 1989; **29**: 265–271.
22. Zannis VI, Kurnit DM, Breslow JL. Hepatic Apo-A-I and Apo-E and intestinal Apo-A-I are synthesized in precursor isoprotein forms by organ cultures of human fetal tissues. *J Biol Chem* 1982; **257**: 536–544.
23. MacIntyre SS, Kushner I, Samols D. Secretion of C-reactive protein becomes more efficient during the course of acute phase response. *J Biol Chem* 1985; **260**: 4169–4173.
24. Baumann H, Isseroff H, Latimer JJ, Jarheis GP. Phorbol ester modulates interleukin-6 and interleukin-1 regulated expression of acute phase plasma proteins in hepatoma cells. *J Biol Chem* 1988; **263**: 17390–17396.
25. Muñoz E, Beutner U, Zubiaga A, Huber BT. IL-1 activates two separate signal transduction pathways in T helper type II cells. *J Immunol* 1990; **144**: 964–969.

ACKNOWLEDGEMENTS. Brigitte Janvier, Geneviève Marret and Patricia Gouache are gratefully acknowledged for their skilful technical assistance. This work was supported by grant CRE No. 910307 from Institut de la Santé et de la Recherche Médicale (INSERM).

**Received 17 June 1992; accepted in revised form 30 July 1992**

## References

[PDF_00385] Andus T., Geiger T., Hirano T., Kishimoto T., Tran-Thi T. A., Decker K., Heinrich P. C. (1988). Regulation of synthesis and secretion of major rat acute-phase proteins by recombinant human interleukin-6 (BSF-2/IL-6) in hepatocyte primary cultures.. Eur J Biochem.

[PDF_00434] Baumann H., Isseroff H., Latimer J. J., Jahreis G. P. (1988). Phorbol ester modulates interleukin 6- and interleukin 1-regulated expression of acute phase plasma proteins in hepatoma cells.. J Biol Chem.

[PDF_00378] Birch H. E., Schreiber G. (1986). Transcriptional regulation of plasma protein synthesis during inflammation.. J Biol Chem.

[PDF_00420] Chomczynski P., Sacchi N. (1987). Single-step method of RNA isolation by acid guanidinium thiocyanate-phenol-chloroform extraction.. Anal Biochem.

[PDF_00368] Davis R. A., Dluz S. M., Leighton J. K., Brengaze V. A. (1989). Increased translatable mRNA and decreased lipogenesis are responsible for the augmented secretion of lipid-deficient apolipoprotein E by hepatocytes from fasted rats.. J Biol Chem.

[PDF_00406] Delers F., Mangeney M., Raffa D., Vallet-Colom I., Daveau M., Tran-Quang N., Davrinches C., Chambaz J. (1989). Changes in rat liver mRNA for alpha-1-acid-glycoprotein, apolipoprotein E, apolipoprotein B and beta-actin after mouse recombinant tumor necrosis factor injection.. Biochem Biophys Res Commun.

[PDF_00384] Dinarello C. A. (1984). Interleukin-1.. Rev Infect Dis.

[PDF_00394] Edbrooke M. R., Burt D. W., Cheshire J. K., Woo P. (1989). Identification of cis-acting sequences responsible for phorbol ester induction of human serum amyloid A gene expression via a nuclear factor kappaB-like transcription factor.. Mol Cell Biol.

[PDF_00410] Feingold K. R., Serio M. K., Adi S., Moser A. H., Grunfeld C. (1989). Tumor necrosis factor stimulates hepatic lipid synthesis and secretion.. Endocrinology.

[PDF_00398] Ganter U., Arcone R., Toniatti C., Morrone G., Ciliberto G. (1989). Dual control of C-reactive protein gene expression by interleukin-1 and interleukin-6.. EMBO J.

[PDF_00380] Gauldie J., Richards C., Harnish D., Lansdorp P., Baumann H. (1987). Interferon beta 2/B-cell stimulatory factor type 2 shares identity with monocyte-derived hepatocyte-stimulating factor and regulates the major acute phase protein response in liver cells.. Proc Natl Acad Sci U S A.

[PDF_00413] Grunfeld C., Adi S., Soued M., Moser A., Fiers W., Feingold K. R. (1990). Search for mediators of the lipogenic effects of tumor necrosis factor: potential role for interleukin 6.. Cancer Res.

[PDF_00416] Guillouzo A., Delers F., Clement B., Bernard N., Engler R. (1984). Long term production of acute-phase proteins by adult rat hepatocytes co-cultured with another liver cell type in serum-free medium.. Biochem Biophys Res Commun.

[PDF_00431] Macintyre S. S., Kushner I., Samols D. (1985). Secretion of C-reactive protein becomes more efficient during the course of the acute phase response.. J Biol Chem.

[PDF_00366] Mahley R. W. (1988). Apolipoprotein E: cholesterol transport protein with expanding role in cell biology.. Science.

[PDF_00372] Mangeney M., Cardot P., Lyonnet S., Coupe C., Benarous R., Munnich A., Girard J., Chambaz J., Bereziat G. (1989). Apolipoprotein-E-gene expression in rat liver during development in relation to insulin and glucagon.. Eur J Biochem.

[PDF_00437] Muñoz E., Beutner U., Zubiaga A., Huber B. T. (1990). IL-1 activates two separate signal transduction pathways in T helper type II cells.. J Immunol.

[PDF_00388] Oliviero S., Cortese R. (1989). The human haptoglobin gene promoter: interleukin-6-responsive elements interact with a DNA-binding protein induced by interleukin-6.. EMBO J.

[PDF_00423] Piccoletti R., Aletti M. G., Bernelli-Zazzera A. (1986). Inflammation-associated events in liver nuclei during acute-phase reaction.. Inflammation.

[PDF_00391] Poli V., Cortese R. (1989). Interleukin 6 induces a liver-specific nuclear protein that binds to the promoter of acute-phase genes.. Proc Natl Acad Sci U S A.

[PDF_00375] Ribeiro A., Mangeney M., Cardot P., Loriette C., Rayssiguier Y., Chambaz J., Bereziat G. (1991). Effect of dietary fish oil and corn oil on lipid metabolism and apolipoprotein gene expression by rat liver.. Eur J Biochem.

[PDF_00401] Rogers J. T., Bridges K. R., Durmowicz G. P., Glass J., Auron P. E., Munro H. N. (1990). Translational control during the acute phase response. Ferritin synthesis in response to interleukin-1.. J Biol Chem.

[PDF_00404] Tu G. F., De Jong F., Apostolopoulos J., Nagashima M., Fidge N., Schreiber G., Howlett G. (1987). Effect of acute inflammation on rat apolipoprotein mRNA levels.. Inflammation.

[PDF_00428] Zannis V. I., Kurnit D. M., Breslow J. L. (1982). Hepatic apo-A-I and apo-E and intestinal apo-A-I are synthesized in precursor isoprotein forms by organ cultures of human fetal tissues.. J Biol Chem.

